# Supplementary material for: Cross-boundary subsidy cascades from oil palm degrade distant tropical forests
Source: Nat Commun. 2017 Dec 20;8:2231. doi: 10.1038/s41467-017-01920-7 (PMC5738359; doi:10.1038/s41467-017-01920-7)
Supplement: Supplementary file 1 — Supplementary Information [file 41467_2017_1920_MOESM1_ESM.pdf]

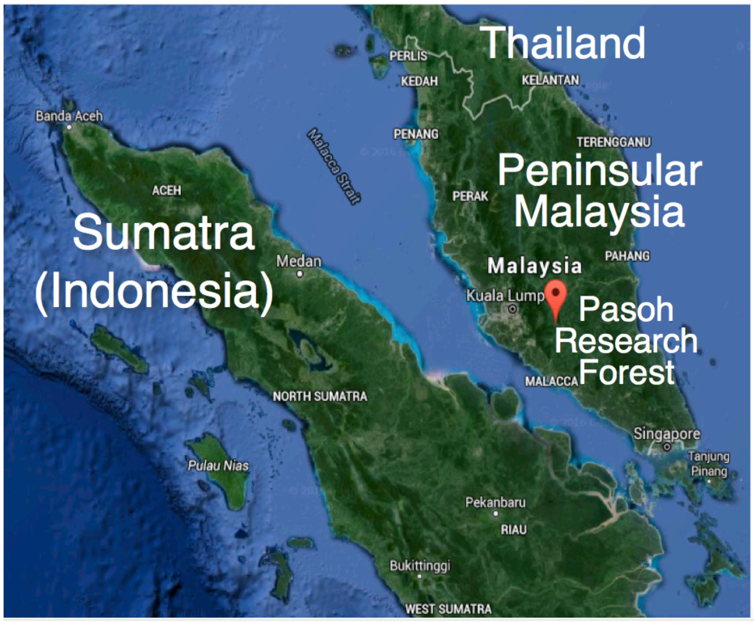

Supplementary Figure 1 l Maps of study site. (**a**) Location of study site within Southeast Asia with the Pasoh Research Forest indicated by the red pointer (Map data: Google, DigitalGlobe).

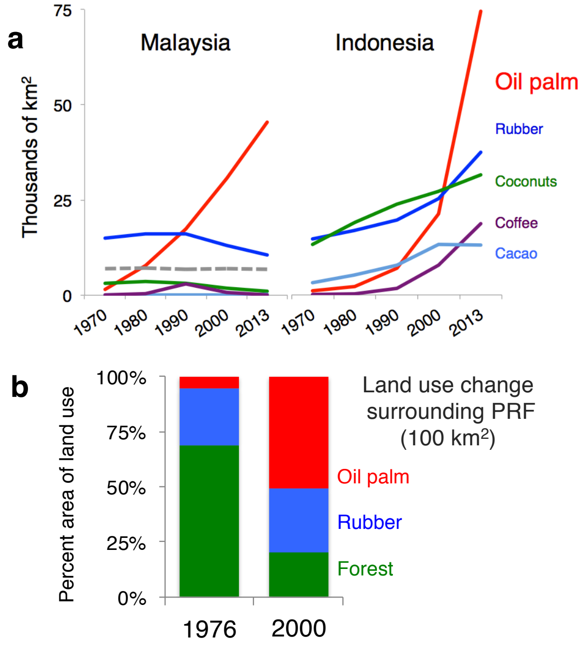

Supplementary Figure 2 l Land use change in Southeast Asia. (**a**) Area of plantation crop expansion in Malaysia and Indonesia from 1970-2013 (data from FAOSTAT). Dashed grey line is tea. (**b**) Land use change in the 100 km^2 area surrounding the PRF research site (from *Naoki et al 2001*).

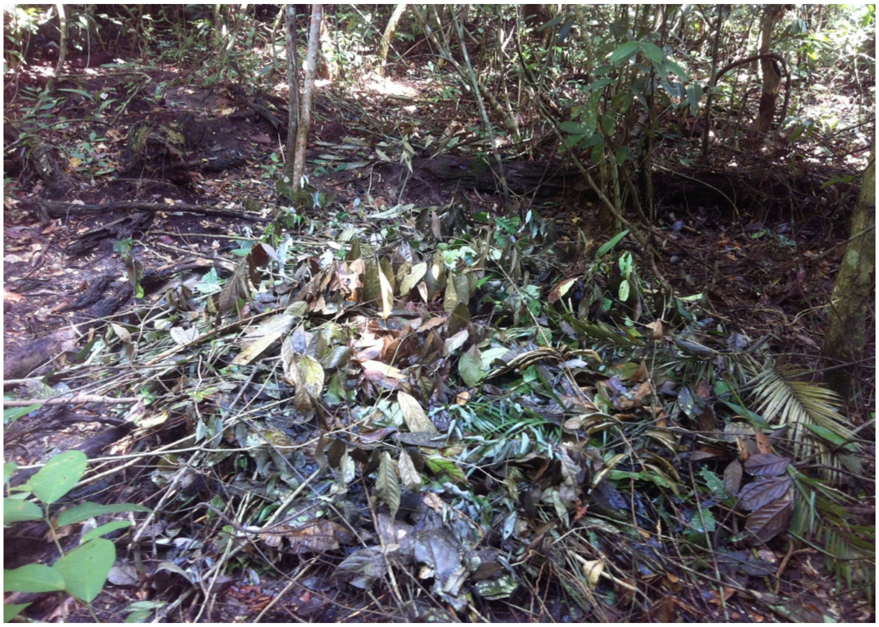

Supplementary Figure 3 | Wild boar nest. Photo of a wild boar nest at the PRF (Photo credit: M.S. Luskin).

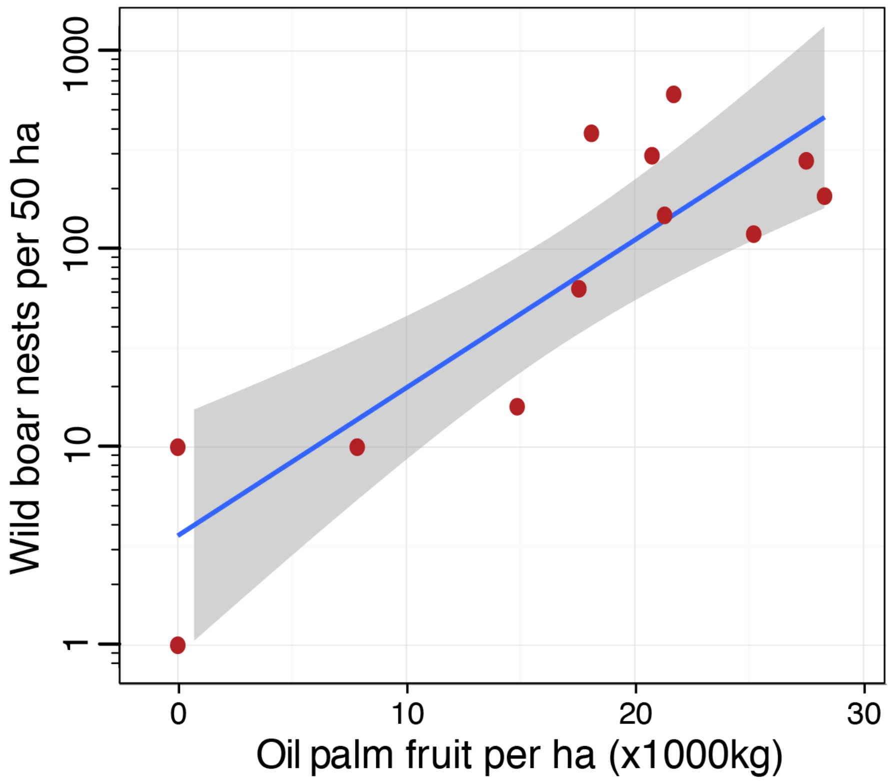

Supplementary Figure 4 l Relationship between oil palm fruit and wild boar nests in adjacent forest. Red dots show years with data on wild boars nests in the 50-ha FDP (located >1.3 km from any forest edge). Oil palm fruit values show the mean of present and previous year production in tone per ha in the plantations adjacent to the PRF. Linear regression line shown in blue [$F_{1,10} = 28.34$, $R^2_{(adj)} = 0.7131$, $P < 0.001$] (Supplementary Table 2), with shaded area showing the 95% confidence interval.

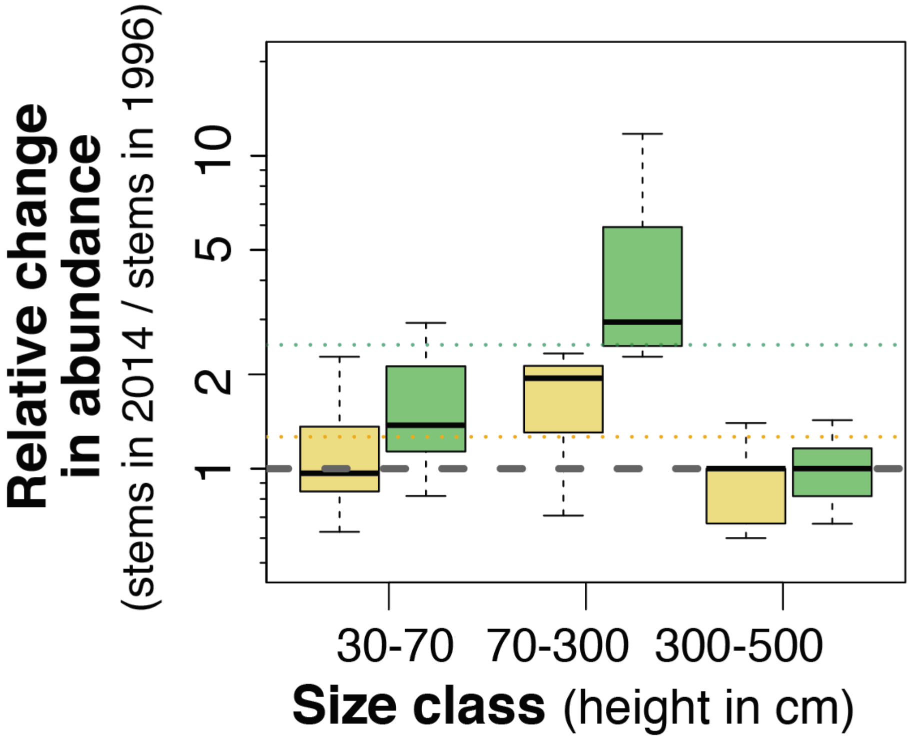

Supplementary Figure 5 l Distinctive impacts from wild boar on the forest understory tree community (30-500 cm height). Size-specific shifts in stem abundances in wildlife exclosures (green) compared to open control plots from 1996 to 2014 (yellow) (*n* = seven replicates of 25 m^2 exclosure plots and paired controls). Colored dashed lines show group means. Whiskers represent distance from upper and lower quartiles to largest and smallest non-outliers. For stems 70-300 cm that are used in wild boar nests, abundance decreased 78.38% in controls compared to exclosures (LRT: $\chi^2_2 = 9.6216$, $P = 0.008$ comparing models with and without a size class * treatment interaction term).

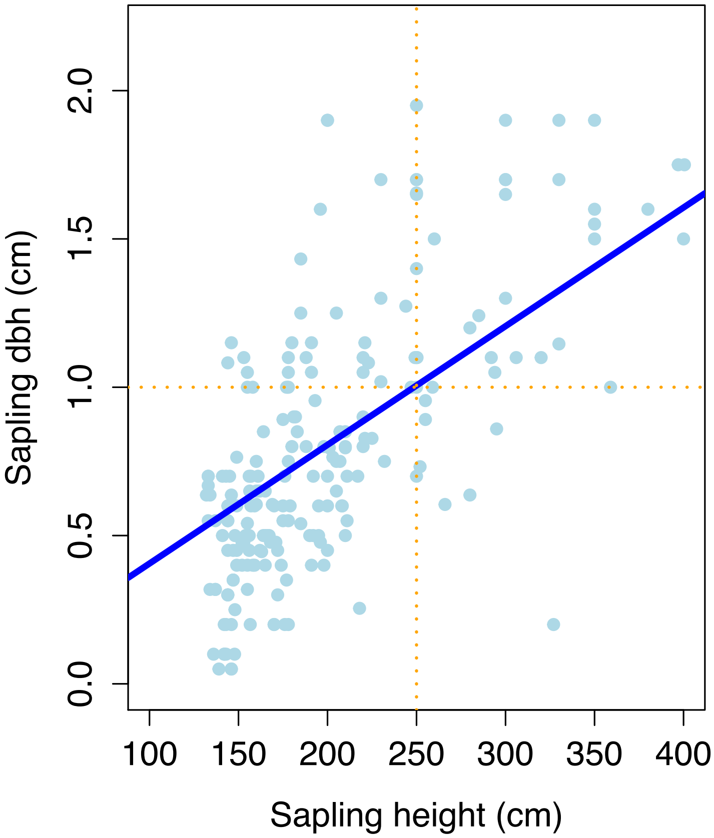

Supplementary Figure 6 | Relationship between tree sapling height and dbh in open control plots in 2014. Results from the exclosure experiment were presented by height, while results from the 50-ha FDP were presented by dbh. This figure is meant to aid comparing results between these two data sources. Orange dotted lines show mean height at 1 cm dbh (249 cm) whereupon saplings would first enter the 50-ha FDP tree census data.

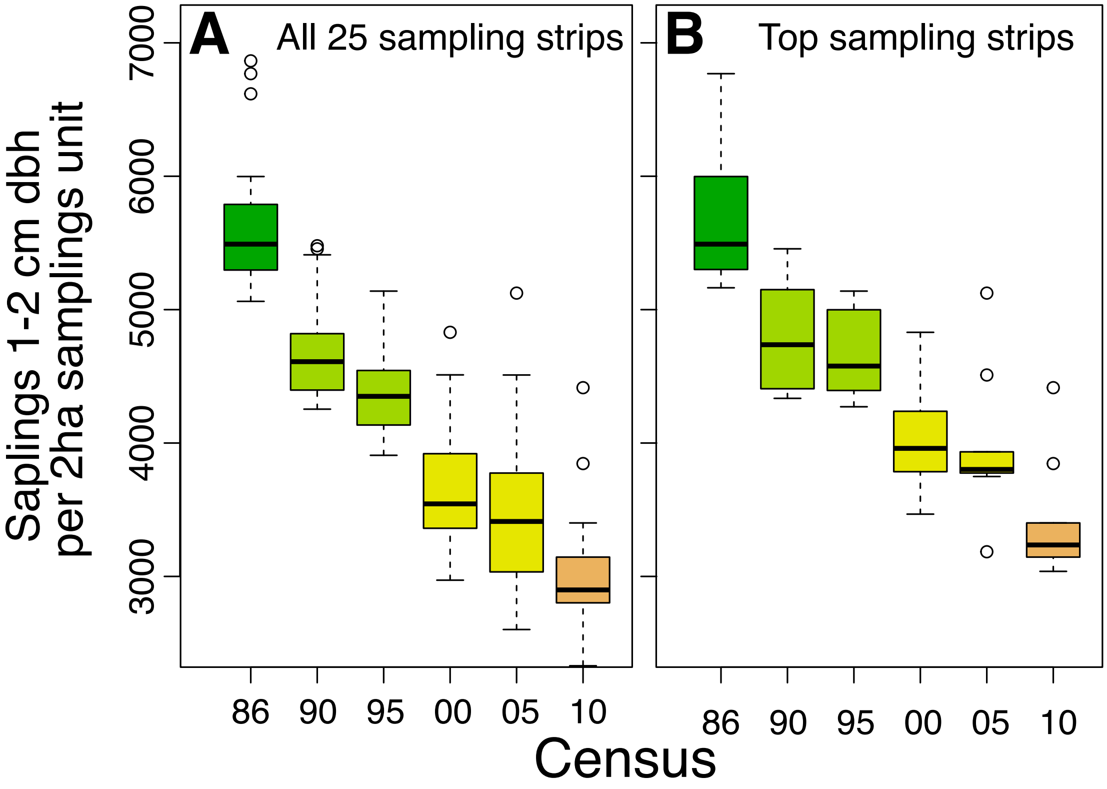

Supplementary Figure 7 l Sapling abundances from 1986-2010 in the PRF 50-ha FDP and potential sampling bias. (**a**) Abundance of saplings 1-2 cm dbh for each of the PRF 50-ha Forest Dynamics Plot censuses shown for each of the 25 sampling units. Sampling units are 2 ha each, surveyed by a team along 20 m X 1000 m strips. (**b**) Abundance in the nine samplings units with the most saplings in 2010. The sampling units with the most saplings are the least likely to suffer from lower sampling effort in 2010 that could explain stem reductions. Boxplot whiskers represent distance from upper and lower quartiles to the mean and open circles are outliers that fall beyond upper or lower quartiles. Colors denote different censuses, used to make comparisons between (a) and (b) easier.

Supplementary Table 1 | Wildlife species in the PRF from 1995-2000 and 2010. Information for 1995-2000 and 2010 was collected from interviews and transects conducted by K. Ickes and camera trapping and transects conducted by M. Luskin, respectively.

Species	Status from 1995-2000	Status in 2010
**Large herbivores/omnivores (>100 kg)**		
Asian elephant *(Elephos maximus*)	Extinct	Extinct
Javan rhinoceros *(Rhinoceros sondaicus)*	Extinct	Extinct
Tapirs (*Tapirus indicus*)	Rare	Common
Sambar deer (*Rusa unicolo*r)	Absent	Rare
Sumatran rhinoceros (*Dicerorhinus sumatrensis*)	Extinct	Extinct
**Medium herbivores/omnivores (10-100 kg)**		
Muntjak barking deer (Muntiacus muntjak)	Rare	Rare
Bearded pig *(Sus barbatus)*	Extremely rare	Extinct
Malayan sun bear *(Helarctos malayan)*	Absent	Rare
Wild pig *(Sus scrofa)*	Hyperabundant	Hyperabundant
**Small herbivores/omnivores (<10 kg)**		
Lesser mouse-deer *(Tragulus kanchil)*	Common	Common
Greater mouse-deer *(Tragulus napu)*	Absent	Rare
Malayan porcupine (*Hystrix brachyura*)	Rare	Common
Binturong *(Arctictis binturong*)	Extremely rare	Rare
Pangolin (*Manis javanica*)	Extremely rare	Extremely rare
Brush-tailed porcupine (*Atherurus macrourus*)	Extremely rare	Extremely rare
**Large predators (>10 kg)**		
Tiger *(Panthera tigris)*	Absent	Absent*
Clouded leopard *(Neofelis nebulosa)*	Absent	Extremely rare
Leopard *(Panthera pardus)*	Absent	Rare
Dhole (*Cuon alpine*)	Extremely rare	Extremely rare
Marbled cat (*Neofelis marmorata*)	Extremely rare	Extremely rare
**Small predators (<10 kg)**		
Leopard cat (*Prionailurus bengalensis*)	Unknown	Common
Yellow-throated marten (*Martes flavigula*)	Extremely rare	Rare
Short-tailed mongoose *(Herpestes brachyurus*)	Unknown	Rare
Flat-headed cat (*Prionailurus planiceps*)	Unknown	Extremely rare
**Primates**		
Pig-tailed macaque (*Macaca nemestrina*)	Hyperabundant	Hyperabundant
White-handed Gibbon *(Hylobates lar)*	Common	Common
Banded Leaf Monkey (*Presbytis melalophos*)	Common	Common
Dusky Leaf Monkey (*Presbytis obscura*)	Common	Common
Long-tailed macaque *(Macaea jascicu/aris*)	Unknown	Uncommon
Siamang (*Hylobates syndaetylus*)	Rare	Rare

**There was one confirmed presence of a tiger in 2007 that remained <1 month.*

Supplementary Table 2 | Model results of relationship of wild boar with oil palm fruit (shown in Fig. S5). Regression model results for wild boar nests in the 50-ha PRF as a function of oil palm fruit production (tonnes ha^{-1}) in adjacent plantations (mean of last year and present year). Linear regression preformed in R.

Coefficients

Term	Estimate	Std. Error	t value	Pr(>ltl)
(Intercept)	0.4914	0.2833	1.7350	0.1135
Oil palm (2-yr mean)	0.0773	0.0147	5.2720	0.0004

Residual standard error: 0.4528 on 10 degrees of freedom
Multiple R^2 = 0.7354, $R^2_{(adj)}$ = 0.709
F-statistic: 27.8 on 1 and 10 DF, P = 0.0003615

Supplementary Table 3 | Model results for exclosure experiment (shown in Fig. 3B). The change in relative stem density from 1996 to 2014 (RC) in control plots and fenced exclosures as a function stem size (Sz) and the proportion of broken stems in 2014 (PB14). Height size classes were 30-70 cm (Sz30), 70-300 cm (Sz70) and 300-500 cm (Sz300). Mixed effects used to include a random block effect (Rep.Group) for each exclosure and paired controls. Analysis conducted using the lme4 package in R with linear mixed model fit by REML (REML criterion at convergence: 8.2) and t-tests conducted with Satterthwaite approximations to degrees of freedom from 'lmerMod' package (N = 7 groups and 42 measurements (three size classes in each paired replicate group). Mixed model $R^2_{(marg)}$ were calculated using 'piecewiseSEM' package [49]. The $R^2_{(marg)}$ was 0.5156.

Coefficients

Term	Parameter	Std. Error	df	t value	Pr(>ltl)
Fixed effects					
(Intercept at Sz30)	0.3351	0.0899	37	3.726	0.0006
Sz70	0.5452	0.1158	37	4.708	3.46E-05
Sz300	-0.5116	0.1449	37	-3.53	0.0011
PB14	-0.9825	0.2710	37	-3.626	0.0009
Sc300:PB14	1.1927	0.3722	37	3.204	0.0028
Random effects:		***Std.Dev.***			
Replicates (σ_b^2)	8.62e-17	9.28e-9			
Residual (σ_ϵ^2)	0.0595	0.2438			

Supplementary Table 4 | Model results relating abundance shifts in the 50-ha FDP to wild boar nests (shown in Fig. 4a). Change in relative density of saplings during the five 50-ha FDP census periods was evaluated as a function of wild boar nests and sapling size (1-10 cm dbh). We included a dummy term ('Saplings <1.5') to test if there were distinct shifts in the smallest size class (1-1.5 cm dbh), and interaction term (Wild boars * Saplings <1.5) to test if these shifts were related to wild boar nest abundance. The model also includes variables to account for negative density dependent mortality (Dens_Dep), size-specific mortality (Size), directional drift through time (Time), and temporal autocorrelation between censuses (Temporal_Corr).

Term	Description
Response	Abundance change over 5-yr census intervals
Sapling dbh	Continuous variable denoting sapling dbh
Saplings <1.5	Categorical variable for stems in smallest size class (cm dbh)
Year	Years since 1986; continuous
Wild boar	Mean wild boar nests in the 50-ha FDP during the census period<br>*Modeled using the regression presented in Supplementary Table 2 in order to interpolate for years without data points<br>+log10 scale
Dens_Dep_Sz	Density of same-sized stems at the start of the census interval<br>*Relative to density of stems in the same size in 1986
Dens_Dep_All	Density of all saplings (<10 cm dbh) at the start of the census interval<br>*Relative to density in 1986
Temporal_Corr	Change during previous 5-yr census interval

Coefficients from the top model

Term	Parameter	Std. Error	t value	Pr(>|t|)
(Intercept)	0.974	0.475	2.049	0.048
Wild boar	0.050	0.015	3.318	0.002
Saplings <1.5 cm	0.295	0.100	2.943	0.006
Year	-0.011	0.004	-2.907	0.006
Sapling dbh	0.116	0.042	2.752	0.009
Dens_Dep_Sz	0.455	0.163	2.798	0.008
Dens_Dep_All	-1.535	0.420	-3.659	0.001
Temporal_Corr	-0.209	0.063	-3.322	0.002
Interactions				
Wild boar * Saplings <1.5	-0.175	0.038	-4.598	4.84E-05
Sapling dbh * Dens_Dep_Sz	-0.097	0.038	-2.579	0.014
Sapling dbh * Time	-0.0006	0.0003	-1.865	0.070

Residual standard error: 0.03071 on 37 degrees of freedom
Multiple R^2: 0.7655, $R^2_{(adj)}$: 0.7021
F-statistic: 12.08 on 10 and 37 DF, $P = 6.58e^{-09}$
